# Triggering receptor expressed on myeloid cells − 1 (Trem-1) on blood neutrophils is associated with cytokine inducibility in human *E*. *coli* sepsis

**DOI:** 10.1186/1746-1596-8-24

**Published:** 2013-02-15

**Authors:** Tobias van Bremen, Daniel Drömann, Karin Luitjens, Christoph Dodt, Klaus Dalhoff, Torsten Goldmann, Bernhard Schaaf

**Affiliations:** 1 Medical Clinic III, University Hospital of Schleswig-Holstein, Campus Lübeck, Lübeck, 23538, Germany; 2 Präklinik, Medical Clinic München-Bogenhausen, München, 81925, Germany; 3 Clinical and Experimental Pathology, Research Center Borstel, Airway Research Center North (ARCN), Member of the German Center for Lung Research, Borstel, 23845, Germany; 4 Medical Clinic Nord, Clinic Dortmund, Dortmund, 44145, Germany

**Keywords:** Triggering receptor expressed on myeloid cells − 1 (Trem-1), Sepsis, Hyporesponsiveness, Pattern recognition receptors (PRR), Neutrophils, Endotoxin tolerance

## Abstract

**Background:**

Bacterial sepsis induced immunsuppression via antigen hyporesponsibility increases the risk of nosokomial infections and mortality. Pattern recognition receptors (PRR) might have a central role in the pathophysiology of hyporesponsibility.

**Methods:**

In this study we evaluated in a human *E*. *coli* sepsis cohort, the role of PRR including TLR’s and Trem-1. Expression of Trem-1, TLR2, TLR4, CD14 and HLA-DR on blood monozytes and neutrophils were examined using flow cytometry from 22 patients with E. coli sepsis and 6 healthy controls. LPS and LTA stimulated TNF alpha, IL-10, IL-8 and IL-6 production was studied in a 24 h whole blood assay. Free cytokine serum concentration of TNF alpha, PCT and IP-10 were evaluated.

**Results:**

We found a significant higher expression of Trem-1 and TLR-2 on monocytes and neutrophils in patients compared to healthy volunteers. TLR2 expression (p < 0.05) was higher and HLA-DR lower (p < 0.05) on monocytes of patients with severe sepsis compared to patients with simple sepsis. Trem-1 expression was tendentially higher (p = 0,07) on monocytes and lower on neutrophils of patients with severe sepsis. Trem-1 expression on neutrophils was associated with the IL-10 (LPS: r = 0,61, p < 0.02) and TNF-α inducibility (LPS: r = 0,78, p < 0,002). In addition Trem-1 expression on neutrophils shows a negative correlation to the serum levels of TNF alpha (r = −0,63; p < 0,005), IP-10 (r = −0,5; p < 0,035) and procalcitonin (r = −0,59; p < 0,007).

**Conclusions:**

Patients with *E*. *coli* sepsis are characterized by an association of Trem-1 expression on blood neutrophils with cytokine inducibility. The TREM-1 pathway on neutrophils might play a role in producing an adequate inflammatory and bactericidal response in bacterial sepsis.

**Virtual Slides:**

The virtual slide(s) for this article can be found here: http://www.diagnosticpathology.diagnomx.eu/vs/4441869398748313

## Background

Despite development in medicine, severe sepsis is a common cause of admission to intensive care units with mortality up to 54% reaching the third leading cause of death in Germany [1].

Clincally mortality in severe sepsis is dichotomic with mortality due to sequela of septic shock and multiorgan failure in the first days and mortality due to secondary, nosocomial infections later in the course of the disease.

An active response to bacterial antigens via activation of immune cells leading to a cytokine response is necessary for the clearance of invading pathogens, but an uncontrolled excessive production of pro-inflammatory cytokines during infection such as tumor necrosis factor (TNF)-α, seems to be responsible for the clinical manifestation of septic shock and the mortality in the first days [2,3]. Later in sepsis an antigen hyporesponsive state, the endotoxin tolerance in combination with reduced apoptosis and reduced proliferative capacity is seen and associated with increased nosocomial second hit infections [4]. Bacterial antigen activates the innate immune system via pattern recognition receptors (PRR’s) on leucocytes and epithelial cells. It has been shown that toll like receptors (TLR’s) and the triggering receptor expressed on myeloid cells − 1 (Trem-1) seem to have a crucial role in immune cell activation in infectious and autoimmune diseases [5-10]. The secondary immunosuppressive state is caused by the initial antigen challenge. Following the phase of proinflammation, induction of aniinflammatory molecules, such as IL-10, lead to a deactivation of immune cells [2,3]. In a recent sepsis study we were able to show in a late phase of human sepsis (day 3) an association of TLR2 and CD14 expression on monocytes with the cytokine hyporesponsiveness [11]. In addition to the innate immunity with cytokine hyporesponsiveness a decreased activity of the adaptive immunity with reduced HLA-DR expression and antigen presentation is seen in sepsis [12].

The role of Trem-1 in endotoxin tolerance has to be defined [13]. Blockade of Trem-1 with antibodies or SI-RNA before or after endotoxin challenge in experimental murine infection influences the proinflammatory reaction [14]. Incomplete antibody blockade or inhibition of Trem-1 signalling reduced mortality without inhibiting bacterial clearance [14,15], but complete blockade of Trem-1 with SI-RNA decreased bacterial clearance and increased mortality [16].

In this study we evaluated in a human *E*. *coli* sepsis cohort, the role of PRR including

TLR’s and Trem-1. We hypothesized that Trem-1 expression on neutrophils and/or monocytes associated with endotoxin tolerance in severe bacterial sepsis.

The endotoxin hyporesponsiveness was tested in a whole blood stimulation assay. We investigated the TLR2, TLR4, CD14, HLADR and Trem-1 expression on blood neutrophils and monocytes of patients with *E*. *coli* sepsis and healthy controls. To have an insight into the functional activity of the receptor we correlated Trem-1 expression on neutrophils and monocytes in sepsis patients with the cytokine release after stimulation with LPS and LTA.

## Methods

### Sepsis patients

A total number of 22 patients with a positive blood culture for *Escherichia coli* and sepsis (defined according to [17]) were investigated in a prospective manner. Patients below 18 years or with defined immunodeficiency (hematologic or solid neoplasia, glucocorticoid or cytotoxic therapy, HIV infection or immunoglobulin deficiency) were excluded from the study.

The source of sepsis was the urinary tract (n = 20), the lung (n = 1) and the gastrointestinal (n = 1). 17 out of 22 (77%) of the patients had a predisposing chronic disease (pulmonary disease, cardiovascular disease, neurologic disease, renal insufficiency, diabetes mellitus).

### Control group

Six unrelated healthy persons, all of white origin without signs of inflammatory disease, served as a control group. The study has been performed in compliance with the Declaration of Helsinki and with the approval of the ethics committee of the University Lübeck (AZ: 04-157). Written informed consent was obtained from patients or their relatives and healthy volunteers

### Study protocol

Venous blood samples were obtained once in healthy controls. In patients a blood sample was taken when a positive blood culture for *E coli* was reported by the microbiologist (24 to 48 hours after clinical diagnosis of sepsis).

### Sepsis severity

Severe sepsis was defined as sepsis with organ dysfunction according to Bone et al. [17]. Septic shock was defined as sepsis in combination with sepsis induced systolic blood pressure of < 90 mmHg for at least 30 min, in the absence of other causes of shock, and at least 4 h of inotropic support after adequate fluid replacement [14]. For group comparison, patients with simple sepsis were compared with patients with severe sepsis including patients with septic shock and/or multiorgan failure (MODS). In addition the acute physiology score (APS), the APACHE II Score (including APS) and clinical/laboratory parameters were evaluated.

### PMN and PBMC purification and flow cytometry

30 ml of blood was obtained by venepuncture and collected into sterile heparinized tubes. PBMC were isolated by Bicoll/Ficoll density gradient centrifugation. PBMCs were cultured in 24-well tissue plates (Biochrome, Berlin, Germany) using endotoxin-free RPMI 1640 medium (Biowhittaker, Belgium) supplemented with 2 mM L-glutamine (Gibco, Eggenstein, Germany) at a density of 0.5 × 10^6^ cells/ml at 37°C in a 5% CO_2_ humidified atmosphere for a period of 3 h. The expression of Trem-1, TLR2, TLR4 ,HLA-DR and CD14 on monocytes and TLR2, TLR-4 and Trem-1 on neutrophils was determined using a fluorescence activated cell sorter (FACS Calibur, Becton Dickinson, Heidelberg, Germany). Data acquisition and analysis were performed with CellQuest software (Becton Dickinson, Heidelberg, Germany). Each measurement contained ≥ 10,000 cells in the monocyte population determined by characteristic forward/orthogonal light scattering in a density plot. PBMC (1 × 10^6^) were incubated on 4°C with 5 μl of following antibodies: anti-Trem-1, -TLR2, -TLR4, -CD14 and –HLA-DR or isotype control (eBioscience, San Diego, USA). The expression of surface markers was calculated as mean fluorescence intensity (MFI) since no bimodal distribution was found.

### Whole blood stimulation

Whole blood stimulation assay was done as described previously [18]. In brief, 2,5 mL of heparinized blood was diluted 1:10 with RPMI 1640 (Biochrome, Berlin, Germany) supplemented with Pen/Strep 1% (Gibco, Germany) and immediately stimulated with 1 μg/mL LPS from Escherichia coli serotype 026:B6 (Sigma, St. Louis, USA) or with 100 μg/ml LTA from *Stapylococcus aureus* (Sigma L2515, St. Louis, USA). Samples were incubated in PPN tubes at 37°C with 5% CO_2_. Each experiment also included controls without LPS or LTA. Cell-free supernatants were removed after 24 h and stored at −80°C until assayed.

### Cytokine assay

IL-6, IL-8, IL-10 and TNF-α measurement of supernatant from whole blood stimulation and from unstimulated sera was performed using commercially available enzyme-linked immunosorbent assay kits (Biosource, Solingen, Germany).

Measurement of soluble Trem-1 (R&D Systems) and Procalcitonin (Acris Antibodies, Germany) was performed by enzyme-immuno-assay.

### Statistics

Nonparametric statistics were used throughout the study. Data are given as mean ± SD. The Wilcoxon signed rank test was used for comparison of paired samples, for comparisons of independent samples the Mann–Whitney-U-test was used. Correlations were made with the Spearman’s rank correlation. Calculations were carried out with Statistica for Windows, version 5, 1997. A p value of < 0.05 was considered significant.

## Results

### Demographic data

Demographic data and parameter of sepsis severity (APACH II, APS, septic complications and mortality) are shown in Table 1. Mean age of the 22 patients was 72 years. Ten patients were male, 12 patients female. Simple sepsis was seen in 11 patients, severe sepsis in 6, septic shock in 4 and MODS in 1 patient (Table 1).

**Table 1:** Demographic and clinical data of 22 patients with E coli sepsis

**Age(mean** **+** **SD in years)**	72 ± 4,24
**Male**	10
**Sepsis severity**	
**Sepsis**	11
**severe sepsis**	6
**septic shock**	4
**MODS**	1
**APACHE II** *	16 ± 9,02
**APS** *	8,5 ± 7,3
**Septic complications**	
**Acute renal failure**	5 (22,7%)
**DIC** *	4 (18,2%)
**28 day mortality**	7 (%)

### TLR2, TLR4, CD14, HLA DR and Trem-1 cell expression of sepsis patients compared to healthy controls

**Figure 1:**
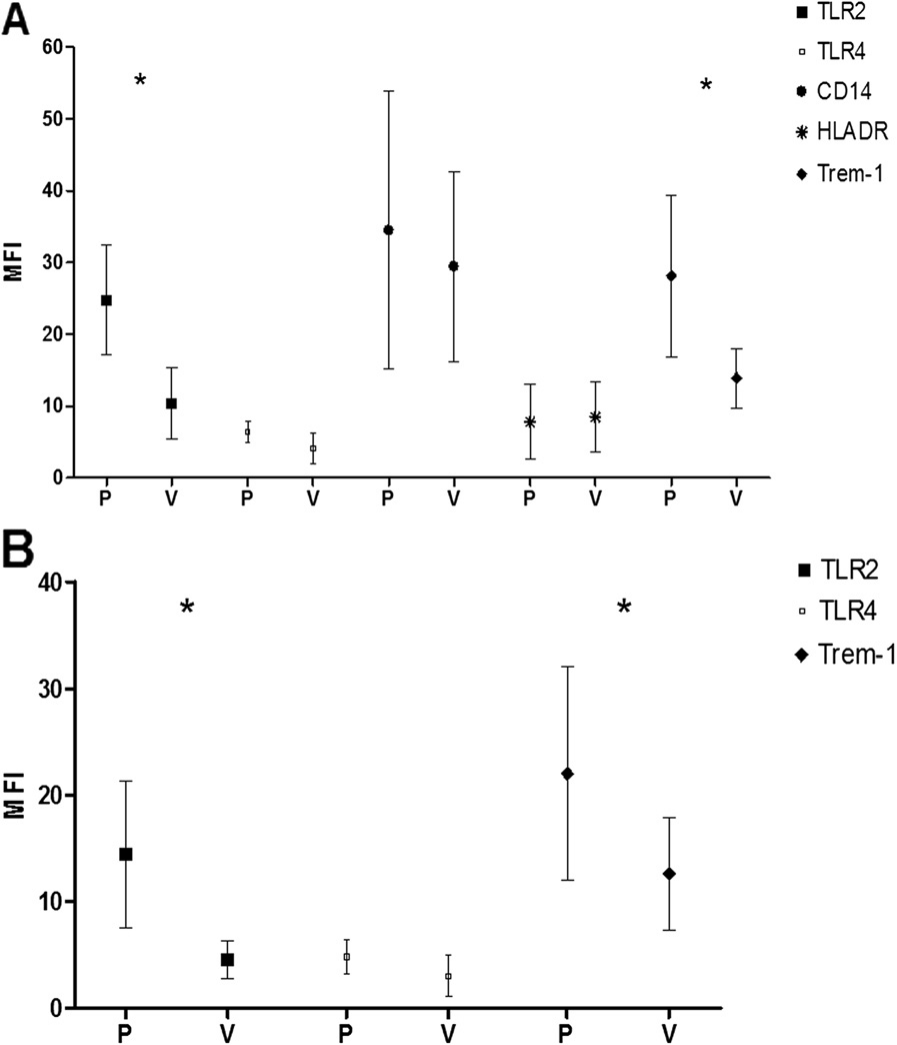
**A: Flow cytometry expression of TLR2, TLR4, CD14, HLA-DR, Trem-1 on monocytes.** MFI ± SD is shown for patients (P) and healthy volunteers (V); * < 0,05; MFI = mean fluorescence intensity. **B**: Flow cytometry expression of TLR2, TLR4, Trem-1 on neutrophils. MFI ± SD is shown for patients (P) and healthy volunteers (V); * < 0,05; MFI = mean fluorescence intensity.

The monocytes expression of Trem-1 and TLR2 was significant higher compared to healthy volunteers (p < 0.05). HLA-DR, CD14 and TLR4 expression showed no significance (Figure 1).

The neutrophil expression of Trem-1 and TLR2 was significant higher compared to healthy volunteers (p < 0.05). TLR4 expression showed no significance (Figure 1).

### TLR2, TLR4, CD14, HLA-DR and Trem-1 expression of sepsis patients according to sepsis severity

The monocyte expression of Trem-1 (trend) and TLR2 (significant) are higher in patients with severe sepsis (including septic shock and MODS) compared to patients with simple sepsis (severe sepsis vs. simple sepsis: Trem-1: 31,47 (±12) vs. 24.54 (±19,65); p = 0,07; TLR2: 27.57 (±8,59) vs. 22,08 (±8,3) p < 0,05) (Table 2).

**Table 2:** **TLR2**, **TLR4**, **HLA**-**DR and Trem**-**1 expression on blood cells according to sepsis severity**

	**Simple sepsis**	**Severe sepsis**	**p**
**Monocytes**			
**TLR2**	22,08 (±8,3)	27,57 (±8,59)	<**0**,**05**
**TLR4**	6,72 (±1,62)	6,0 (±1,38)	>0,05
**Trem**-**1**	24,54 (±19,65)	31,47 (±12)	>0,05
**HLA**-**DR**	10,38 (±5,03)	5,2 (±4,17)	<**0**,**05**
**Neutrophils**			
**TLR2**	14,63 (±6,84)	14,15 (±4,16)	>0,05
**TLR4**	5,15 (±2,09)	4,56 (±0,96)	>0,05
**Trem**-**1**	24,0 (±11,91)	19,5 (±7,33)	>0,05

The monocyte expression of HLA-DR was significantly lower in patients with severe sepsis compared to simple sepsis (5,216 (±4,17) vs. 10,38 (±5,03), p < 0,05).

The neutrophil expression of Trem-1 is tendentially lower in patients with severe sepsis (severe sepsis vs. simple sepsis: Trem-1: 19,5 (±7,33) vs 24,00 (±11,91); p > 0,05).

No correlation was found between TLR-2, TLR-4, CD-14, Trem-1, HLA-DR expression and APS, APACHE II, Serum-CRP and Serum PCT values or death (data not shown).

### Cytokine concentration in unstimulated serum

To evaluate the magnitude of cytokine blood levels during sepsis, serum values of several cytokines and inflammatory markers were evaluated. TNF-α, PCT and soluble Trem-1 (sTREM) values were significantly higher (p < 0.05) in patients with severe sepsis compared to simple sepsis (Table 3).

**Table 3:** Serum levels of TNF-α, PCT soluble Trem-1 (sTREM) and CRP according to sepsis severity

	**Simple sepsis**	**Severe sepsis**	**p**
**TNF**-**α(pg/ml)**	5,62 (±1,91)	28,18 (±15,23)	<**0**,**05**
**PCT(ng/ml)**	9,05 (±15,65)	80 (±89,4)	<**0**,**05**
**sTrem**-**1(pg/m)l**	347,9 (±182,1)	741 (±446,9)	<**0**,**05**
**CRP(mg/l)**	153 (±27,21)	219 (±30,93)	>0,05

### Cytokine inducibility in whole blood stimulation

To test the cytokine inducibility and a possible hyporesponsiveness during sepsis, a whole blood assay with LPS and LTA *in vitro* stimulation was used. Hyporesponsiveness for LPS stimulation was seen in patients with severe sepsis compared to simple sepsis patients for IL6, IL8, IL10 and TNFα reaching significant results for TNFα (Table 4).

**Table 4:** Cytokin inducibility in whole blood stimulation for IL-6, Il-8, Il-10 and TNF-α

		**Simple sepsis**	**Severe sepsis**	**p**
**IL**-**6**				
	LPS	14253 ± 10714	7200 ± 6823	> 0,05
	LTA	15513 ± 9166	8871 ± 12318	> 0,05
**IL**-**8**				
	LPS	1515 ± 1026	1217 ± 1405	> 0,05
	LTA	4735 ± 2925	2002 ± 2096	> 0,05
**IL**-**10**				
	LPS	15,55 ± 14,13	12,47 ± 11.83	> 0,05
	LTA	33,83 ± 17,85	13,27 ± 12,50	> 0,05
**TNF**-**α**				
	LPS	760,6 ± 745,4	325,2 ± 623,8	< 0,01
	LTA	1530 ± 1820	239,5 ± 381,5	< 0,01

### Functional activity of Trem-1 on neutrophils

To test the functional activity of TLR2, TLR4, CD14 and Trem-1, the cytokine inducibility after LPS and LTA stimulation was correlated with the receptor expression.

**Figure 2:**
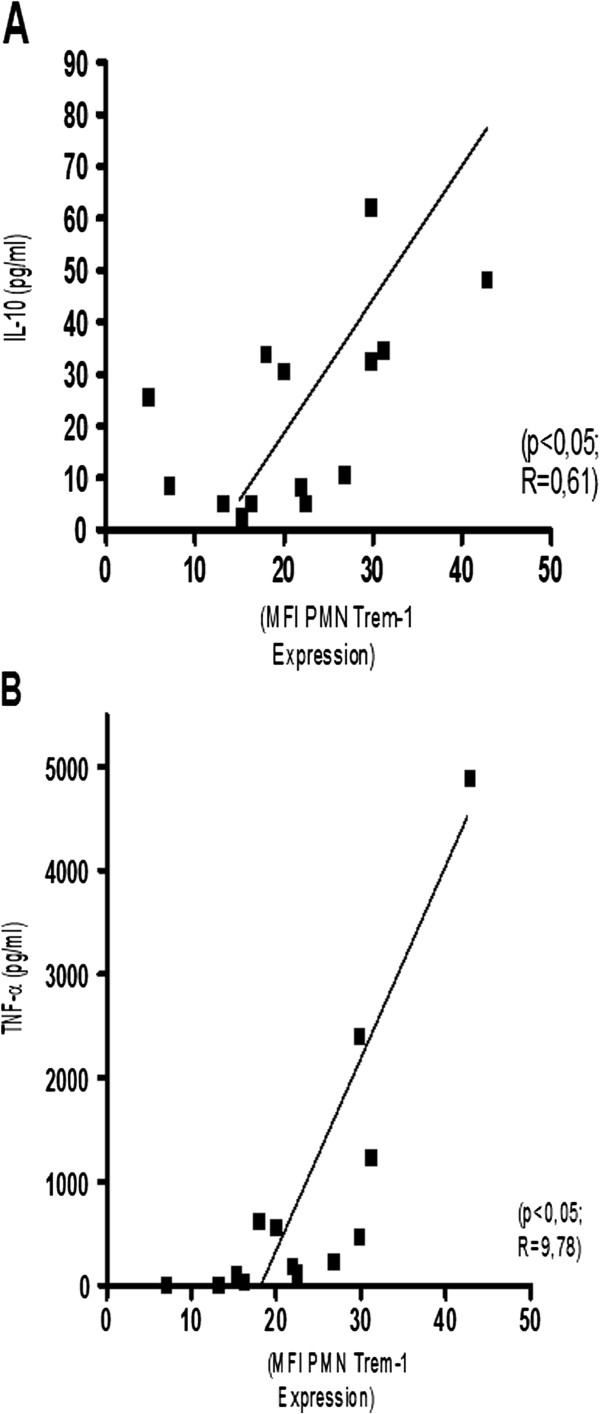
**A: Correlation between Trem-1 expression on neutrophils and LPS induced inducibility of IL10. B**: Correlation between Trem-1 expression on neutrophils and LPS induced inducibility of TNF-α.

In sepsis patients Trem-1 expression on neutrophils was associated with the IL-10 (LPS: r = 0,61, p < 0.02) and TNF-α inducibility (LPS: r = 0,78, p < 0,002) (Figure 2). No correlation was found for the cytokine inducibility with the expression of Trem-1, TLR2, TLR4, CD14 or HLA-DR on monocytes (data not shown).

### Association of trem-1 expression on neutrophils with immune activation

**Figure 3:**
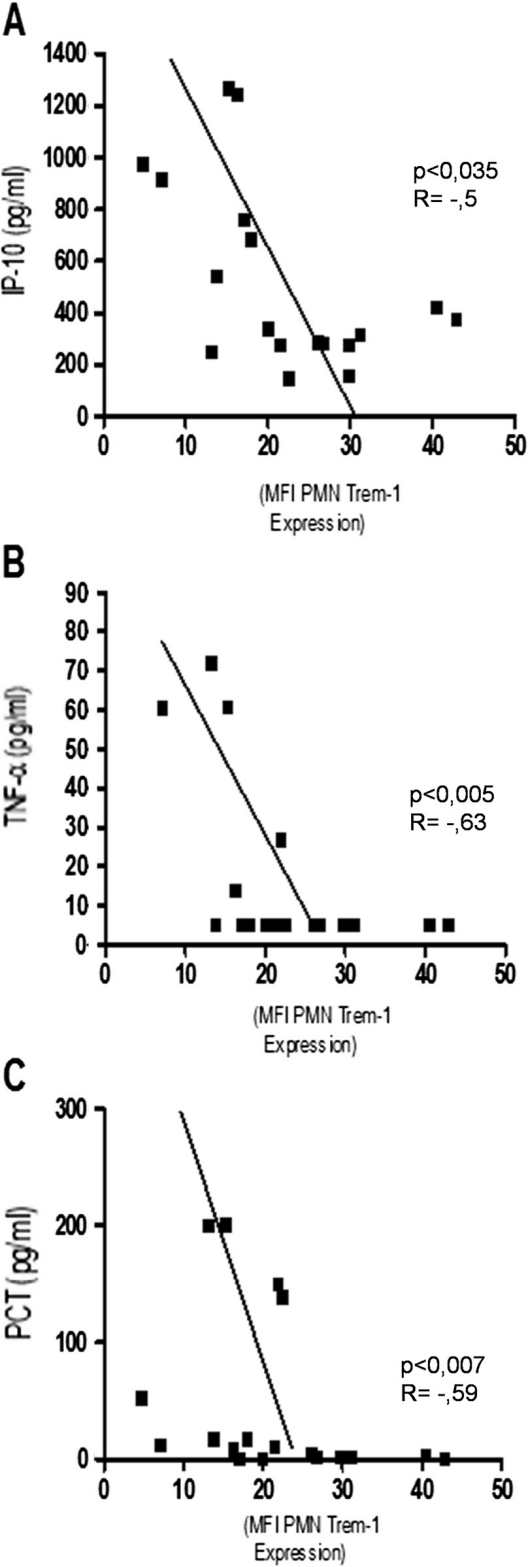
**A: Correlation between neutrophil Trem-1 expression and serum IP-10 concentration (p < 0,035, R = −0,5). B**: Correlation between neutrophil Trem-1 expression and Serum TNFα concentration (p < 0,005, R = −0,63). **C**: Correlation between neutrophil Trem-1 Expression and Serum PCT concentration (p < 0,007, R = −0,59).

In sepsis patients Trem-1 expression on neutrophils shows a negative correlation to the serum levels of TNF-α (r = −0,63; p < 0,005), IP-10 (r = −0,5; p < 0,035) and procalcitonin (r = −0,59; p < 0,007) (Figure 3). No correlation was found for IL-10.

No correlation was found for the cytokine serum levels with the expression of Trem-1, TLR2, TLR4, CD14 or HLA-DR on monocytes (data not shown).

## Discussion

Our study demonstrates increased expression levels of TLR2, TLR4, CD14 and Trem-1 protein expression on blood monocytes of patients with *E*. *coli* sepsis.

An interesting association was found for the Trem-1 expression on blood neutrophils with the cytokine production: Low Trem-1 expression on neutrophils was associated with low cytokine inducibility after stimulation with bacterial antigen. Trem-1 receptor on neutrophils might be involved in the induction of cytokines during sepsis [19]. Low Trem-1 expression on neutrophils might be associated with hyporesponsiveness during severe human sepsis.

The host defence needs to detect invasion of pathogenic organism to induce an inflammatory response in order to control the infection. Pathogen recognition receptors (PRR) as TLR’s and CD14 are activated via pathogen associated molecular pattern, namely LPS for gram negative and LTA for gram positive bacteria. The activation of the immune cells causes via induction of gene transcription the production of cytokines.

Trem-1 is a relatively new discovered receptor expressed on neutrophils and monocytes, also involved in the innate immune response to bacterial pathogens [13,14]. Activation of Trem-1 together with TLR ligands as LPS or LTA causes increased production of TNFα [20,21]. It has been shown that in patients with sepsis and in mice with experimental LPS induced septic shock, Trem-1 is upregulated on neutrophils [14]. In line with this data, Trem-1 was upregulated on neutrophils and monocytes in our study (Figure 1). On the other hand, activation of Trem-1 and TLR4 caused a reduction of the production of the antiinflammtory cytokine IL-10 [21].

In patients with severe *E*. *coli* sepsis we found a trend towards lower Trem-1 on neutrophils but higher soluble Trem-1 (compared to patients with simple sepsis, Table 4). This data are in line with recent data from Oku et al., who have shown reduced Trem-1 on neutrophils but increase soluble Trem-1 in sepsis patients compared to SIRS patients [9]. A correlation between soluble Trem-1 and sepsis severity has also been shown in recent papers [22-24].

Why do we and others find a differential pattern in sepsis with high soluble Trem-1 and low neutrophil-surface TREM-1? Monocyte surface Trem-1 is shed by a metalloprotease [25]. Pina et al. speculate that surface TREM-1 might also be shed from neutrophils explaining low surface Trem-1 in severe sepsis [25].

Neutrophil surface Trem-1 might be involved in the capacity to produce cytokines. We found a positive correlation between Trem-1 expression on neutrophils and cytokine inducibility. Low Trem-1 expression on neutrophils was associated with low cytokine production after stimulation. In addition we found a negative correlation of Trem-1 on neutrophils and markers of immune activation. The higher levels of TNF-α in serum were found, the lower Trem-1 expression on neutrophils was measured. Since surface Trem-1 is activated early during sepsis [14] and downregulated during severe sepsis, a negative loop might exist.

Our result might be interesting for the phenomenon of endotoxin hyporesponsiveness during sepsis [26,27]. Sepsis can induce monocyte hyporesponsiveness, causing an anergic state of the immunesystem to gram negative and gram positive bacteria [26]. Patients with hyporesponsiveness are immunosuppressed, and are prone to secondary infections associated with increased morbidity and mortality [26-28]. In our study we can show a hyporesonsiveness in severe sepsis seen as reduced TNF inducibility (Table 4) to *E*. *coli* endotoxin and to *S*. *aureus* antigen (lipotheichonic acid, LTA). Since reduced surface Trem-1 on neutrophils was associated with reduced TNF inducibility, surface Trem-1 on neutrophils might be involved in hyporesponsiveness.

In addition to above data, as shown before, HLA-DR expression on monocytes was decreased in severe sepsis, probably influencing the capacity of antigen presentation [29].

Beside a negative effect with immunosuppression, some data show a protective effect of Trem-1 inhibition via reduction of the overwhelming toxic proinflammation during septic shock. Bouchon et al. were able to show a protective effect of antibody blockade of Trem-1 in Mice [14]. Injection of antibody 1 hour before endotoxin challenge was protective and caused reduction in mortality from 94 to 24%. The same effect was seen in experimental *E*. *coli* sepsis in mice [14]. The effect was less pronounced when antibodies were given after the endotoxin injection. On the other hand complete silencing of Trem-1 via SI-RNA decreased bacterial clearance and increased mortality in a mouse model of infection [16]. Partial inhibition of Trem-1 in the bacterial peritonitis model produced a significant survival benefit [16]. Downregulation of the cytokine production during severe human sepsis might have a protective effect reducing the dangers of overwhelming cytokine production in the beginning and a detrimental effect later on since immunosuppression increase the risk of secondary infections [30]. Hyporesponiveness might protect the person of overwhelming cytokine production but increases the risk of secondary infections.

Our study has several limitations. The parameters were only measured at sepsis diagnosis (transversal study), serial evaluation (longitudinal study) was not performed. Since the study population was small, more subjects are required for future trials. Newly discovered factors (e.g. Trem-2, Trem-like transcript 1 and 2) might also be involved.

## Conclusions

Patients with severe *E*. *coli* sepsis compared to patients with simple sepsis are characterized by endotoxin tolerance and cytokine hyporesponsiveness.

Trem-1 on blood neutrophils is tendentially lower in patients with severe sepsis, associated with reduced cytokine inducibility.

Since Trem-1 has been discussed as a potential therapeutic target in sepsis [30], the precise role in inflammatory response to bacterial infection of soluble and surface bound Trem-1 on neutrophils and monocytes should be further evaluated in clinical and experimental studies.

## Competing interests

The authors declare that they have no competing interests.

## Authors’ contributions

TB and KL carried out the flow cytometry and were involved in the design of the study and drafting the manuscript. BS, KD, TG, CD, JR and DD conducted the clinical part of the study and were involved in the design and coordination of the study and drafting the manuscript. All authors read and approved the final manuscript.
